# Twelve-month results of cyanoacrylate closure in patients with venous leg ulceration

**DOI:** 10.1016/j.jvsv.2026.102635

**Published:** 2026-10-07

**Authors:** Kathleen Gibson, Manjit Gohel, Ahmed Kayssi, Joseph Sabat, Nicolas Néaume, Leigh Ann O'Banion, Paul Gagne, Misaki Kiguchi, Neil M. Khilnani, Raghu Kolluri, Nicholas Salkowski, Daniel J. Denmark, Alun H. Davies

**Affiliations:** a Division of Vascular Surgery, University of Washington, Seattle, WA; b Department of Vascular Surgery, Cambridge University Hospitals, Cambridge, UK; c Division of Vascular Surgery, University of Toronto, Toronto, Canada; d Vascular Surgery Department, Pima Heart and Vascular, Tucson, AZ; e Interventional Phlebology Centre, Centre Clinique Pasteur, Toulouse, Occitanie, France; f Department of Surgery, University of California, Fresno, CA; g The Vascular Care Group, Darien, CT; h Department of Vascular Surgery, MedStar Washington Hospital Center, Washington, DC; i Division of Vascular and Interventional Radiology, Weill Cornell Medicine, New York Presbyterian Hospital, New York, NY; j OhioHealth, Heart and Vascular Department, and Syntropic Core Lab, Columbus, OH; k Peripheral Vascular Health Department, Medtronic, Minneapolis, MN; l Department of Vascular Surgery, Charing Cross Hospital, Imperial College, London, UK

**Keywords:** Chronic venous disease, Venous leg ulcers, Cyanoacrylates, Safety, Quality-of-life

## Abstract

**Background:**

Venous leg ulceration (VLU) is a major cause of patient distress and health care burden. Previous studies have highlighted the importance of early endovenous ablation of superficial venous reflux to expedite healing and prevent recurrence, but the optimal venous ablation modality is unclear.

**Objective:**

The aim of this study was to evaluate the effectiveness and safety of cyanoacrylate closure in patients with VLU.

**Design:**

A global, prospective, single-arm observational study was conducted in 17 centers across the United States and Europe. A total of 125 participants with VLU were included and treated with the VenaSeal closure system (Medtronic), in addition to standard wound therapy, including multilayered compression. Adjuvant treatments to varicosities, perforators or the subulcer plexus were left to the discretion of treating clinicians. The primary outcome was VLU healing through 12 months. Secondary outcomes included time to ulcer recurrence, anatomic vein closure, target limb adverse events (AEs), and changes in quality-of-life (QoL) measures.

**Results:**

One hundred twenty-five participants with VLU and saphenous vein reflux were enrolled. The mean ± standard deviation age was 67.9 ± 14.76 years, with 38.4% females and an overall mean body mass index of 32.4 ± 9.35. Concomitant foam sclerotherapy treatments were performed in 19.7% (31/157) of target veins. At 12 months, ulcer healing rate was 81.3%, and ulcer recurrence rate was 17.0%. The median time to ulcer healing was 92 days (95% confidence interval, 74-137). Anatomic vein closure was successful, with a closure rate of 86.6% among all treated veins through 12 months. Clinical events committee-adjudicated AE and serious AEs included: two granulomas, one hypersensitivity reaction, and four cases of phlebitis of the treated limb. Health care services used outside the index procedure yielded 0.12 adjunctive therapies per vein-year. Venous-specific and generic QoL scores showed sustained improvement compared with baseline scores.

**Conclusions:**

VenaSeal effectively eliminated saphenous reflux in patients with VLU. The ulcer healing rate was high, and the recurrence rate was low. Complication rates were also low and QoL improved.

**Article Highlights**

• **Type of Research:** Multicenter, prospective, single-arm cohort study
• **Key Findings:** One hundred twenty-five participants with advanced chronic venous insufficiency and various other comorbidities were treated with cyanoacrylate closure to observe the impact on chronic and large ulcers. The ulcer healing rate was 81.3% through 1 year. Safety events were independently adjudicated. Duplex scans reveal anatomic vein closure (86.6%) through 1 year.
• **Take Home Message:** Cyanoacrylate closure is impactful for lasting ulcer healing in ailing participants.

Venous leg ulceration (VLU) represents an advanced manifestation of chronic venous disease (CVD) and is associated with substantial morbidity and significant health care resource utilization worldwide.^1^, ^2^, ^3^ Comprehensive treatment to the entire great saphenous vein (GSV) has demonstrated optimal wound healing in Clinical, Etiology, Anatomy, and Pathophysiology (CEAP) class 6 patients, irrespective of the modality.^4^, ^5^

Compared with the broader CVD population, patients with VLU are older, have more comorbidities, and more extensive superficial venous reflux.^2^, ^6^ They also more commonly experience unintentional weight loss, fatigue, reduced gait speed, lower physical activity, and are more likely to have reflux extending into or involving below-knee saphenous vein trunks.^6^, ^7^ Nonthermal endovenous interventions may therefore be particularly advantageous in this population, as they avoid the pain of tumescent anesthesia and the risk of thermal nerve injury associated with treatment of below-knee saphenous segments, facilitating treatment of the entire refluxing saphenous vein in a single session. Cyanoacrylate closure (CAC) is a catheter-based, nonthermal, and nontumescent technique for vein closure with excellent technical success.^6^, ^8^, ^9^, ^10^ Although case series and retrospective studies have shown encouraging results after CAC in patients with VLU, prospective data remains limited.^11^, ^12^, ^13^ Given the frailty and sarcopenia often seen in this population, effective treatment is particularly important.^6^, ^7^ The aim of this global, multicenter noncomparative study was to evaluate the effectiveness and safety of CAC in participants with VLU.

## Methods

### Trial design and objectives

A prospective, single-arm, observational study conducted across 17 centers in six countries, enrolled 125 participants with active VLU treated with CAC using the VenaSeal closure system (Medtronic).^14^ Participants had symptomatic CVD with CEAP class 6. The primary end point was time to ulcer healing through 12 months. Secondary end points included time to ulcer recurrence, anatomic vein closure, the proportion of superficial reflux treated, postindex interventional health care utilization, and physician or patient-reported quality of life (QoL) and treatment satisfaction questionnaires. Safety end points were adjudicated by an independent clinical events committee (CEC) through 12 months. This trial was conducted in compliance with Strengthening the Reporting of Observational Studies in Epidemiology guidelines and was registered on ClinicalTrials.gov (NCT03820947).^14^, ^15^ The trial was sponsored by Medtronic, the manufacturer and distributor of VenaSeal.

This study was conducted in accordance with the Declaration of Helsinki and all local regulatory requirements. The protocol was approved by the Institutional Review Board of all participating sites. All participants provided written informed consent.

### Participants

Eligible participants were ≥18 years of age with CEAP class 6 disease and a refluxing saphenous vein segment ≥10 cm in length and ≥3 mm in diameter. Inclusion criteria are outlined in Supplementary Table I (online only) in the Supplementary Materials (online only). Participants were followed for 12 months from procedure date with reported study follow-up visits at 7 and 30 days as well as 6 and 12 months (Fig 1). In addition, visits for active ulcer healing and photography adjudication occurred at 2, 3, 4, 5, 8, and 10 months.

**Fig 1:**
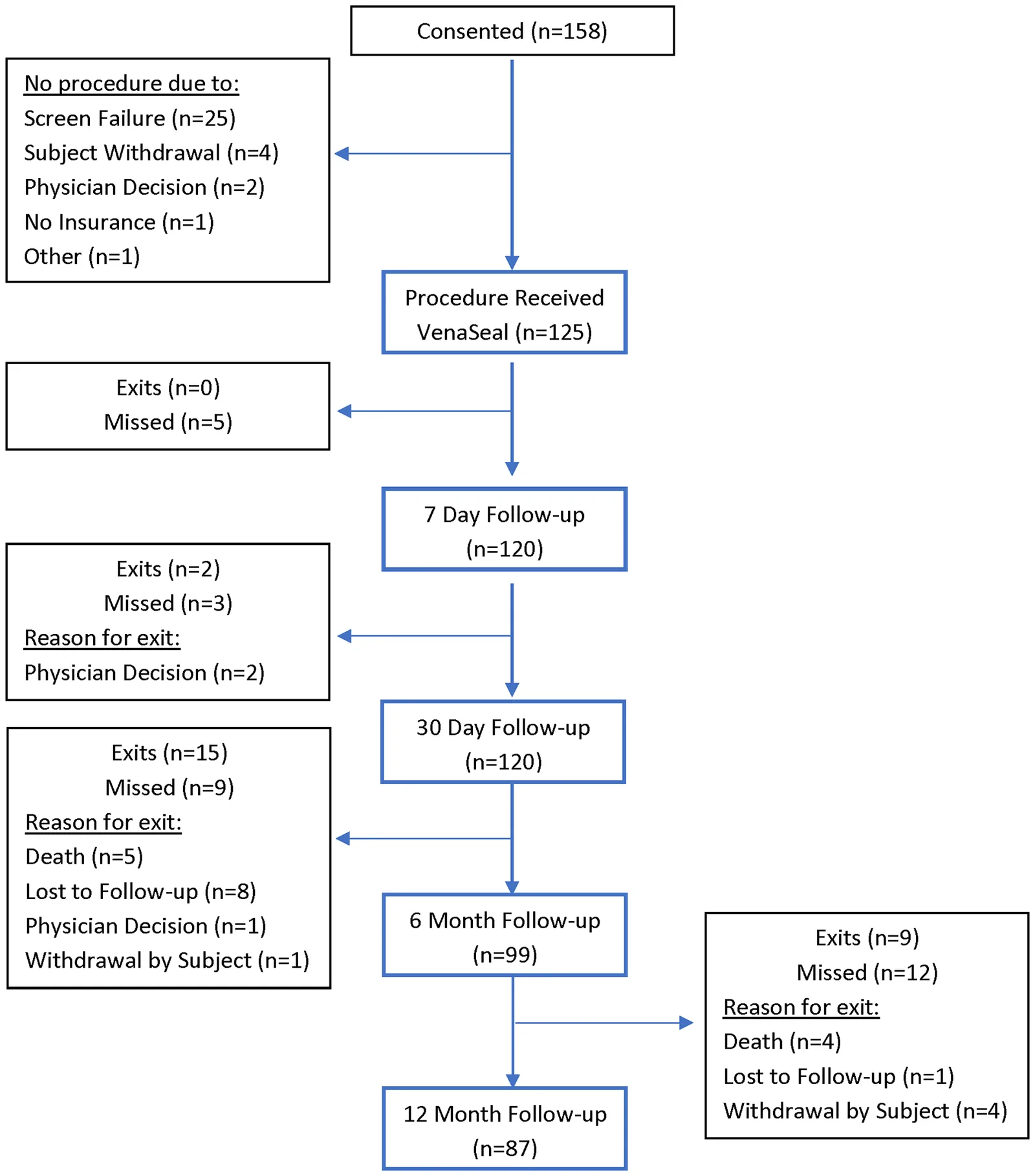
Strengthening the Reporting of Observational Studies in Epidemiology (STROBE) diagram. Participant flowchart in the venous leg ulceration (VLU) cohort of the VenaSeal Spectrum trials. Patients who consented before the procedure but did not receive treatment with VenaSeal were excluded from the study as a screen failure. An additional 59 patients exited after initial consent because of screen failure, death, withdrawal, physician decision, or a loss to follow-up. One patient death that occurred shortly following their 12-month follow-up visit within the visit window is not reflected in this diagram. Participants were enrolled between September 2020 and May 2023.

### Details of intervention

All participants underwent CAC using the VenaSeal system, an *n*-butyl-2-cyanoacrylate adhesive approved for the permanent closure of lower-extremity superficial truncal veins, including GSV, small saphenous vein, anterior accessory saphenous vein (ASV), and posterior ASV. Concomitant sclerotherapy or phlebectomy was permitted during index procedure and left to the treating physician's discretion.^16^ Postprocedure care, including compression, anticoagulation, and analgesia was left to operator discretion, as per local protocols.^17^ Additional adjunctive therapies for target limb varicosities in the ulcer bed were allowed 90 days after index procedure, according to the investigator's site standard of care to continue ongoing wound therapy.^18^

### Outcome measures

The primary end point was time to ulcer healing, verified by an independent core laboratory (Syntropic Core Lab). Secondary outcomes included time to ulcer recurrence, anatomic closure of the primary target vein (TV), target-limb adverse events (AEs), use of adjunctive procedures, return to work or normal activities, and changes in QoL. Elimination of clinically relevant superficial truncal reflux in each TV at the index procedure was measured as the percentage of TV length successfully treated within the GSV, small saphenous vein, ASV, and posterior ASV. Vein closure was defined (site reported) as complete closure of the treated segment on duplex ultrasound, with all discrete segments of patency to be ≤5 cm in length. AEs and serious adverse events (SAE) through 12 months were adjudicated by an independent CEC. Events of interest included: hypersensitivity, phlebitis, granuloma, and ablation-related thrombus extension (ARTE), as well as all SAEs including deep venous thrombosis (DVT) and pulmonary embolism. Hypersensitivity reaction was defined as an allergic reaction in the target limb to the VenaSeal adhesive, including, but not limited to hives, asthma, anaphylaxis, or nonspecific cutaneous and subcutaneous inflammation. Phlebitis was defined as a post-treatment inflammatory reaction involving the treated vein, residual varicosities, or outside of the treatment zone owing to the draining of said vessels; or into cutaneous or subcutaneous tissues located within or outside of the treatment zone and adjacent tissues, due to the treatment. Granulomas were defined as localized inflammatory collections characterized by macrophage aggregation. ARTEs are defined as extensions of a thrombus, extending from the treated vein into the deep venous system, consistent with previous descriptions of endovenous glue- or heat-induced thrombosis.^19^

Health care utilization in this study was defined as health care resource use related to target limb venous reflux disease over 12 months. This included inpatient admissions and hospital nights, outpatient and clinic visits, emergency department visits, and missed workdays, as well as any adjunctive procedures. Time to return to activities was defined as the number of days from the index procedure to the resumption of work or normal daily activities (all participants). QoL was measured with the venous-specific revised Venous Clinical Severity Score (rVCSS) and Aberdeen Varicose Vein Questionnaire (AVVQ), and overall QoL was measured with the EuroQoL Group 5-Dimension Self-Report Questionnaire (EQ-5D-5L), the Short-Form Health Survey (SF-36), Venous Treatment Satisfaction Questionnaire (VenousTSQ)^20^, and the Venous Dependent QoL (VenousDQoL).

### Statistical analysis

Kaplan-Meier (KM) estimates were used to calculate the probability of ulcer healing, anatomic vein closure, and ulcer recurrence. Cumulative incidence curves with KM risk estimates were fit for time to ulcer healing, ulcer recurrence, and a survival curve with KM survival estimates was fit for anatomic vein closure. The median time to ulcer healing was calculated from the KM risk estimate. Return to normal activities and work was also summarized as the percentage of participants returned at the 12-month visit. AEs and SAEs were summarized as the percentage of patients with at least one event, stratified by MedDRA System-Organ Class and Preferred Term. Health care utilization was calculated using incidence rates for both adjunctive procedures and health care visits, where the numerator was the total number of procedures or visits, and the denominator was the total follow-up years through the 12-month visit. Paired changes in QoL metrics were summarized using the mean and standard deviation. No significance tests were conducted. Analyses were performed using either SAS version 9.4.0 (SAS Institute) or R version 4.4.2 (R Foundation for Statistical Computing), and data were summarized using descriptive statistics (for continuous variables) or frequencies and percentages (for categorical variables).

## Results

### Participants, VLU characteristics, and intervention

A total of 125 participants with VLU were treated. In all, 157 TVs were treated at 17 clinical sites across six countries from September 10, 2020, to May 3, 2023. Baseline demographics, venous, and VLU characteristics are summarized in Table I. Obesity (38.4%) and lymphedema (28.0%) were the most common comorbidities (Supplementary Table II, online only). Mean body mass index was 32.4 ± 9.35, and 17.6% of participants had a previous DVT. The mean maximum ulcer wound depth and area at baseline were measured at 0.11 ± 0.12 cm and 7.32 ± 11.58 cm^2^ respectively (N = 110 participants). Most ulcers were located in the medial leg (68.0%, 85/125) with 32.0% (40/125) on the lateral leg.

**Table I:** Baseline and target vein procedural characteristics

Characteristics	N = 125 participants N = 125 primary veins N = 157 all veins
Age, years	67.9 ± 14.76
Female, %	38.40
BMI, kg/m^2^	32.4 ± 9.35
Ulcer duration, mean ± SD, days	519.9 (976.1)
Location	
Medial, %	68.0 (85/125)
Lateral, %	32.0 (40/125)
Ulcer size	
Surface area, mean ± SD, cm^2^	7.32 (11.58)
Depth, mean ± SD, cm	0.11 (0.12)
Volume, mean ± SD, cm^3^	0.84 (1.95)
Preprocedural	
Diameter of target vein, mm	6.1 ± 1.72
Target vein	
ASV	0.8 (1/125)
GSV	78.4 (98/125)
PASV	1.6 (2/125)
SSV	19.2 (24/125)
Intraprocedural characteristics	
Length of saphenous reflux treated, cm	51.9 ± 20.41
Perforating vein treated	8.8 (11/125)
TV reflux elimination, %	90.8 ± 13.21
Concomitant foam sclerotherapy	
Primary target vein (participants), %	20.8 (26/125)
All target veins treated (veins), %	19.7 (31/157)

*ASV*, Anterior saphenous vein; *BMI*, body mass index; *GSV*, great saphenous vein; *PASV*, posterior accessory saphenous vein; *SSV*, small saphenous vein; *TV*, target vein.

Numbers are mean ± SD (N) or % (n/N).

The GSV was the most commonly treated vein (Table I). Median ulcer duration was 169 days (range, 14-6816 days). TVs were concomitantly treated with foam sclerotherapy in 26 participants or 19.7% (31/157) of veins. Adjunctive treatments following the index procedure occurred at a rate of 0.12 procedures per vein-year (N = 19 procedures; Supplementary Table III, online only). No participants underwent subsequent phlebectomy through 12 months of follow-up.

### Primary outcomes

At 12 months, the probability of ulcer healing was 81.3% (Fig 2). Median time to ulcer healing was 92 days. Once ulcer healing was achieved, the probability of ulcer recurrence was 17.0% through 12 months (Supplementary Fig, online only).

**Fig 2:**
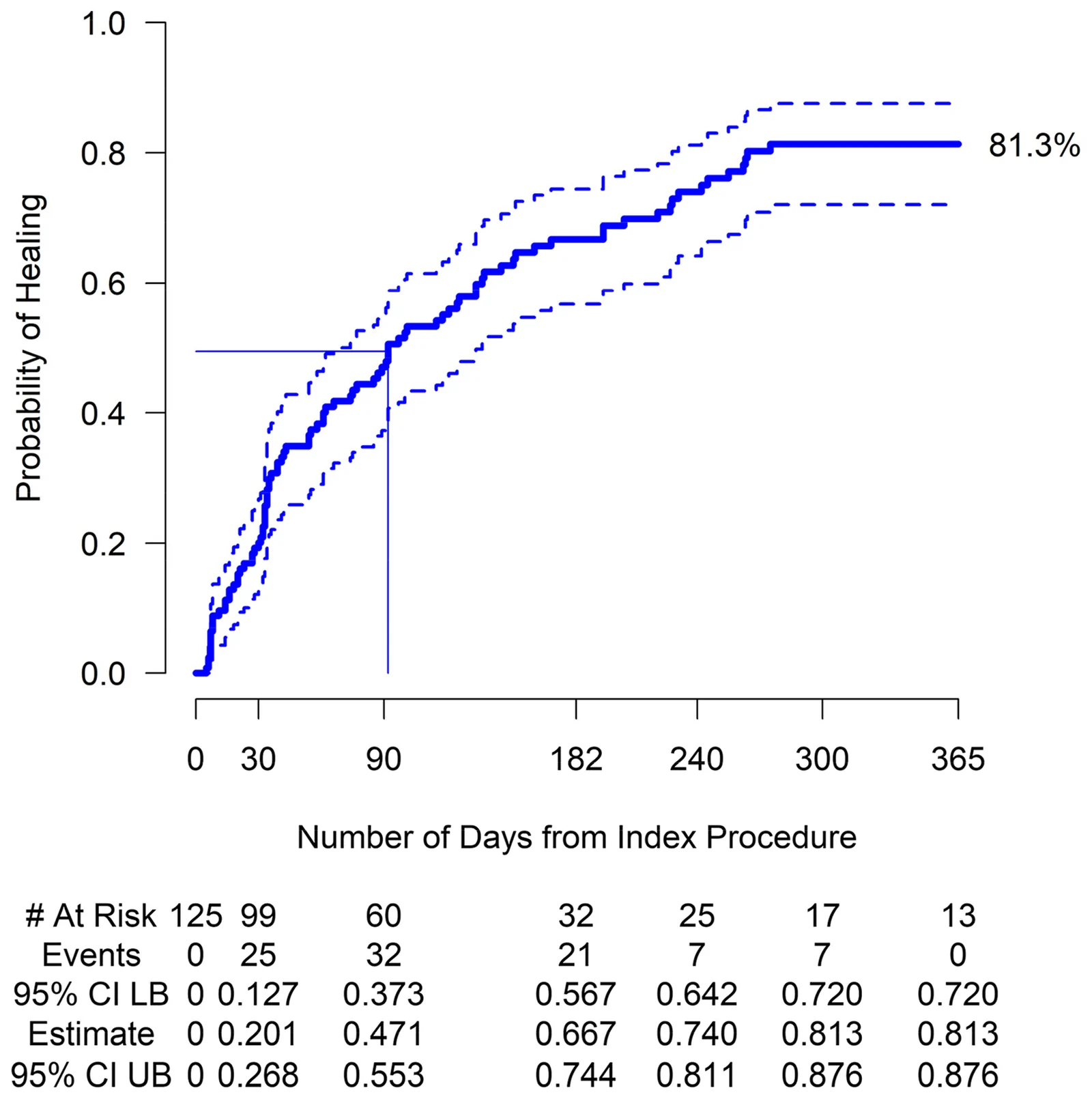
Kaplan-Meier curve—time to ulcer healing. Kaplan-Meier curve demonstrates the probability of wound healing and freedom from progression of ulceration through 12 months in the venous leg ulceration (VLU) cohort of the Spectrum trial. The highlighted point represents the median time to ulcer healing, of which was 92 days [95% confidence interval (*CI*), 74-137].

### Secondary outcomes

The mean length of clinically significant reflux was 51.9 ± 20.41 cm, and the mean proportion of treated superficial reflux was 90.8% ± 13.21%, (values capped at 100%) (Table I; Supplementary Table IV, online only). Some veins did not fully close, but the open veins did not have clinically relevant reflux.

Among the 42 employed participants, two exited the study before 12 months. Of the remaining 40, a total of 30 (75%) returned to normal work duties and 9 (22.5%) returned to lighter duties by 12 months (Supplementary Table V, online only). Return to normal activities was reported by 92.8% (116/125) participants at 12 months. Of these, 69% (80/125) reported no limitations, 30.2% (35/125) reported some limitations, and 2.4% (3/125) had not returned to normal activities. At 90 days, the probability of returning to work was 94.3%, and the probability of returning to normal activities was 87.8%, as calculated using a KM estimate.

Health care utilization after the index procedure included both scheduled and unscheduled encounters. A total of 127 inpatient nights were recorded among 12 study participants, with a mean of 10.6 ± 8.38 nights per hospitalized patient. Outpatient utilization included 51 outpatient visits (0.44 utilizations per person-year), 1355 clinic visits (11.79 utilizations per person-year), and 21 emergency department visits (0.18 utilizations per person-year). Among the 75 patients that missed any work due to subsequent health care utilization, the mean number of workdays missed was 1.25 ± 10.39. Most participants were not employed at baseline (83/125, 66.4%).

QoL and symptom measures including rVCSS, AVVQ, EQ-5D-5L, VenousTSQ, and VenousDQoL SF-36 demonstrated sustained improvement from baseline (Supplementary Table VI and VII, online only). The EQ-5D index score showed the most substantial improvements from baseline to 12 months with an average increase of 0.17. Consistent improvement was reported in the SF-36 physical (increase of 3.80) and mAVVQ scores (decrease by 15.5) from baseline through month 12. The rVCSS decreased by 9.0 through 12 months. The VenousTSQs had minimal changes from 30 days to 12 months (30.9-32.1). The VenousDQoL demonstrated participants' QoL slightly improved through 12 months from baseline, with 9.4% (8/85) available participants reporting an “Excellent” QoL compared with 6.5% (8/123) at baseline.

Probability of anatomic vein closure of the primary TV was 86.6% through 12 months (Fig 3).

**Fig 3:**
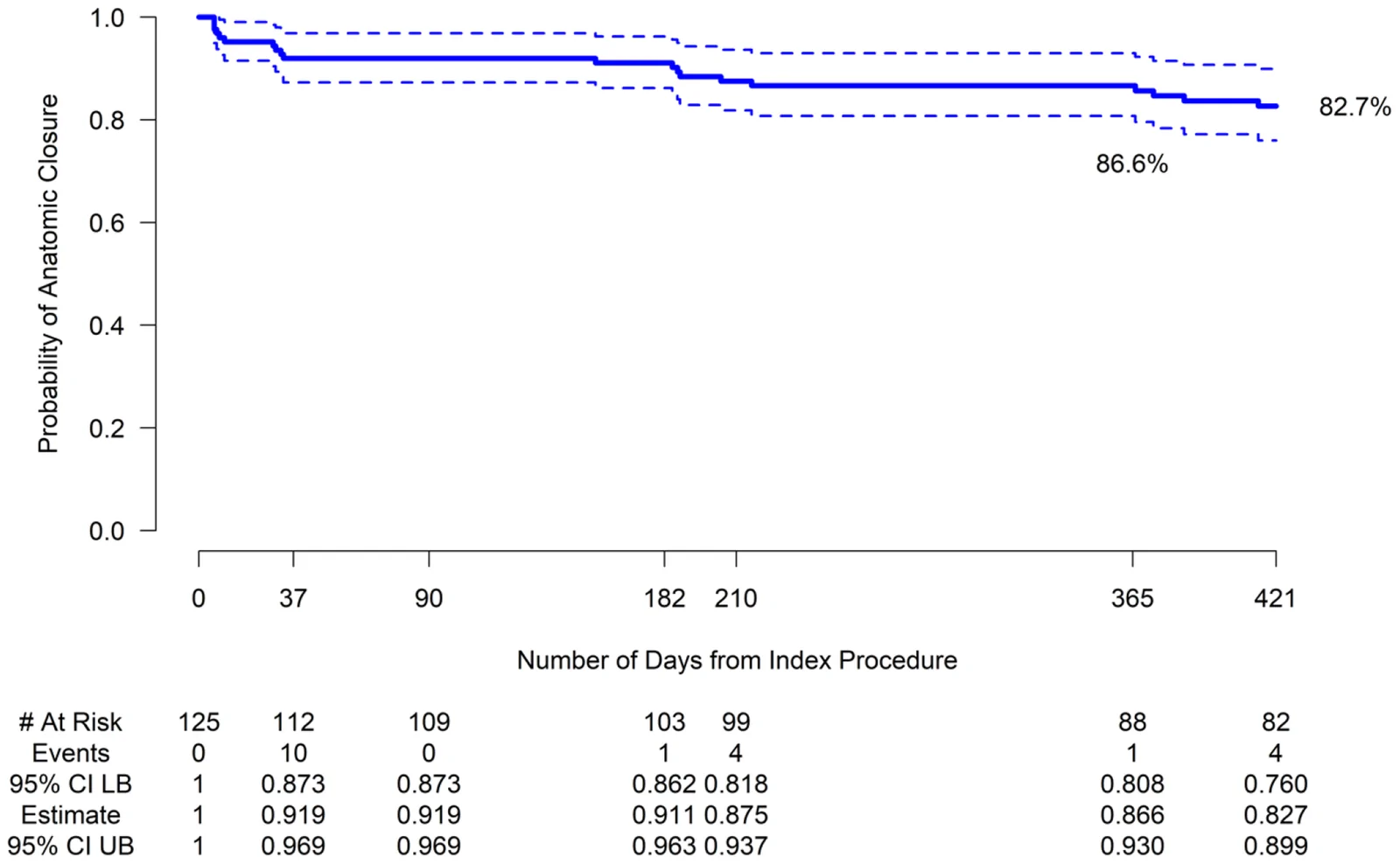
Kaplan-Meier curve—probability of anatomic vein closure of the primary target vein. Kaplan-Meier curve demonstrates the probability of anatomic vein closure of target veins through 421 days from index procedure in the venous leg ulceration (VLU) cohort of the VenaSeal Spectrum trials. At 365 days, the probability of vein closure was 86.6% [95% confidence interval (*CI*), 80.8-93.0] and at 421 days it was 82.7% [95% CI, 76.0-89.9].

### Safety outcomes

AEs adjudicated by the CEC are summarized in Table II. There were 10 participant deaths during the study period; all were adjudicated to be unrelated to the device or procedure. One additional death occurred shortly after the patient's 12-month follow-up visit was completed and is not reflected in the 12-month Strengthening the Reporting of Observational Studies in Epidemiology (STROBE) diagram. One hypersensitivity reaction was reported 6 days after the index procedure, and resolved with compression, oral nonsteroidal anti-inflammatory drugs, and antihistamines within 13 days of onset. Four SAE phlebitis events were reported—three were adjudicated to be causally related to the procedure and two to the study devices. One participant with phlebitis also developed a gastrocnemius vein thrombosis, *Klebsiella* infection, and was resolved in 52 days with antibiotics and compression.

**Table II:** Clinical events committee (CEC) adjudicated adverse events relationships in target limb through 12 months

Event	All	Not related	Causal-study device and/or procedure	Causal-study device	Causal-study procedure
Any adverse events^a^					
Hypersensitivity to VenaSeal Adhesive, % (n/N)	0.8 (1/125)	0.0 (0/125)	0.8 (1/125)	0.8 (1/125)	0.8 (1/125)
Phlebitis, % (n/N)	10.4 (13/125)	1.6 (2/125)	8.8 (11/125)	8.0 (10/125)	8.8 (11/125)
Granuloma^b^, % (n/N)	1.6 (2/125)	0.0 (0/125)	1.6 (2/125)	1.6 (2/125)	1.6 (2/125)
ARTE^c^, % (n/N)	2.4 (3/125)	0.0 (0/125)	2.4 (3/125)	2.4 (3/125)	2.4 (3/125)
Serious adverse events					
Hypersensitivity to VenaSeal Adhesive, % (n/N)	0.0 (0/125)	0.0 (0/125)	0.0 (0/125)	0.0 (0/125)	0.0 (0/125)
Phlebitis, % (n/N)	3.2 (4/125)	0.8 (1/125)	2.4 (3/125)	1.6 (2/125)	2.4 (3/125)
Granuloma^b^, % (n/N)	0.8 (1/125)	0.0 (0/125)	0.8 (1/125)	0.8 (1/125)	0.8 (1/125)
ARTE^c^, % (n/N)	0.0 (0/125)	0.0 (0/125)	0.0 (0/125)	0.0 (0/125)	0.0 (0/125)
Any serious adverse event					
n (events)	72	64	8	4	8
% (n/N)	26.4 (33/125)	24.8 (31/125)	5.6 (7/125)	3.2 (4/125)	5.6 (7/125)
Deaths					
% (n/N)	8.0 (10/125)	8.0 (10/125)	0.0 (0/125)	0.0 (0/125)	0.0 (0/125)

Data are percentage (%) of patients with at least one event [participants (n)/total population (N)] or % (n/N) or number of events. CEC-adjudicated reported data for adverse or severe adverse events through 12 months ± 8 weeks follow-up window.

a Adverse event type and relatedness are adjudicated by an independent CEC.

b CEC-adjudicated adverse event granuloma was reported at the access-site with adhesive protruding from a punctuate ulcer. Serious adverse granuloma was reported below the right knee, 10 cm from the puncture site and accompanied by a *Staphylococcus aureus* infection.

c Ablation-related thrombus extension (ARTE).

Two granulomas were reported before 12 months and were adjudicated as related to the device and procedure. One participant developed a granuloma below the right knee, approximately 10 cm above the puncture site, which required excision under anesthesia. The postexcision course was complicated by recurrent S*taphylococcus aureus* wound infection, treated with two courses of flucloxacillin. Histopathology demonstrated benign thrombus without dysplasia or malignancy, and symptoms resolved in 176 days. Aside from this case, no VenaSeal procedure-related infectious complications were observed. Three ARTE events were reported, two of which had anticoagulants postprocedure. All were adjudicated as device- and procedure-related, and all resolved in 6 to 24 days.

## Discussion

In this prospective multicenter global study of participants with VLUs, treatment with CAC was associated with high ulcer healing rates and improvements in rVCSS and QoL at 12 months. Despite a cohort characterized by large chronic ulcers and a high burden of comorbidities, the probability of ulcer healing at 1 year was 81.3%. The recurrence rate of 17.0% at 1 year is consistent with previous reports.^11^, ^21^, ^22^, ^23^, ^24^, ^25^ Median time to ulcer healing was 92 days, comparable with published randomized controlled trials, including those with smaller baseline ulcer surface areas.^21^, ^23^

QoL improvements in this VLU cohort were more modest than those reported in populations with less advanced disease (CEAP class 1-5), as shown by Xiong et al.^26^ However, the magnitude of change from baseline in our study was similar or greater than previously published studies. In Xiong, et al,^26^ QoL scores improved from 97.33 ± 4.19 (C2-C3) and 94.88 ± 6.56 (C4-C6) at baseline to 12 months. In the Varithena Impact and Efficacy on Wound Healing–Venous Leg Ulcers (VIEW-VLU; NCT03257254) study, Shao et al^22^ reported an increase in EQ-5D-5L QoL by 0.80 through 12 months compared with 0.17 in the present study.

Prospective data on primary TV closure in patients with VLU have been previously reported in the Early Venous Reflux Ablation (EVRA; NCT03286140) trial.^27^ In that study, the primary TV closure rate was 83.3%, although timing was not standardized and was based on a subset of patients with follow-up duplex ultrasound. In the VenaSeal Spectrum VLU study, the 12-month closure rate was 86.6%, slightly higher than EVRA but lower than rates reported in randomized controlled trials of patients with less severe venous disease (C2-C4) treated with VenaSeal, including the VeClose trial, in which 12-month closure rates were 94% to 98%.^9^, ^19^, ^27^, ^28^, ^29^ These differences are most consistent with the more advanced disease seen in the Spectrum VLU CEAP class 6 cohort, including longer-segment reflux, and a higher prevalence of deep venous pathologies characteristic of patients with ulceration.^5^

When considered alongside previous landmark VLU trials, these findings further define the role of intervention in ulcer healing^30^, ^31^. The Effect of Surgery and Compression on Healing and Recurrence (ESCHAR) trial demonstrated that adding surgery for superficial reflux to compression reduced ulcer recurrence but did not shorten time to healing.^30^, ^31^, ^32^ In contrast, the EVRA trial showed that early endovenous ablation, including thermal modalities and sclerotherapy, significantly accelerated healing and increased ulcer-free time in a selected VLU population.^21^, ^23^, ^25^, ^33^, ^34^

The Spectrum VLU study extends these findings to a real-world, global CEAP class 6 cohort, demonstrating that nonthermal, nontumescent adhesive closure with VenaSeal can safely and effectively contribute to VLU healing, with rates comparable to previous studies.^33^ The ability to treat the full length of the refluxing segment, including below-knee disease, without risk of thermal nerve injury, is particularly relevant in this population. Although recurrence rates in Spectrum are similar to those reported in EVRA, more complete treatment of distal reflux may have implications for simplifying the management of recurrent ulcers, as intervention for untreated saphenous segments would not be necessary.^5^, ^18^, ^21^, ^25^, ^35^ Previous trials also lacked detailed characterization of ulcer depth and volume, particularly for circumferential wounds, and the inclusion of those parameters in the present study adds a novel dimension to the VLU evidence base.

Direct comparison between Spectrum and the more selected EVRA populations requires caution given differences in study design, participant selection, demographics, and treatment modalities. However, the 12-month healing rate observed in Spectrum VLU (81.3%) is comparable with that reported in EVRA, despite the inclusion of larger ulcers (>6 cm), longer ulcer duration, and a higher prevalence of comorbid disease in Spectrum.^21^, ^23^, ^25^ Importantly, Spectrum also enrolled participants from a broad mix of global practice settings, including regions with variable access to compression use and vascular care.^34^ This heterogeneity more closely reflects the diversity of patients seen in daily worldwide practice and strengthens the external validity of our findings.

In a previously reported multi-institutional retrospective observational study, the VLU in VenaSeal-treated limbs healed significantly faster than in limbs treated with radiofrequency ablation (median, 43 vs 104 days; *P* = .001), a difference that persisted for both small (<3 cm^2^) and large (>3 cm^2^) ulcers.^11^, ^12^, ^13^ Recurrence and infection rates were similar between groups. Although retrospective and potentially subject to unmeasured confounders, multivariable regressions identified the treatment modality to be the only significant predictor of time to wound healing. These findings are consistent with our results and support the effectiveness of CAC to achieve high healing rates in the VLU population.

Safety outcomes were consistent with previous CAC and VLU studies. Device or procedure-related SAE were extremely low and limited to infection, superficial thrombophlebitis, and rarely, granuloma formation, with rates consistent with previous CAC literature.^13^, ^36^, ^37^, ^38^, ^39^ Serious phlebitis reactions, adjudicated by an independent CEC, occurred in four participants, lower than typically reported.^38^ The hypersensitivity reactions were low (0.8% in this study vs 6.0% in Gibson, et al^37^) and may reflect characteristics of this population, including advanced age. No device-related deep venous thrombotic or embolic events were observed. Despite 17.6% of patients having a history of DVT, ARTE occurred in only one patient with a previous DVT. Though no deaths were attributed to the device or procedure, the mortality rate (8.0%) was higher when compared with the EVRA trial (3.8%) and lower than the ESCHAR trial (17%).^21^, ^25^, ^27^, ^30^, ^31^

The single-arm design is a limitation and precludes direct comparison with other methods of saphenous ablation. This study was conducted during the coronavirus disease-2019 pandemic, which posed challenges for global enrollment and follow-up across participating regions. Sites spanned diverse health care systems and practice environments, improving generalizability but introducing heterogeneity in VLU care.^2^ The decision to forgo a randomized design was intentional to focus on the use of CAC in the VLU population, as prospective data in ulcer patients have been limited.^14^ Outcomes may also reflect the experience of participating physicians and centers. Conversely, the lower (86.6%) closure rates and participants available for follow-up through 12 months (87/99) in this VLU cohort likely relate to the overall disease burden on comorbidities of the study population.

## Conclusions

In summary, saphenous ablation with VenaSeal CAC can be performed safely and effectively in a complex, global CEAP class 6 population, achieving high ulcer healing rates and durable vein closure without tumescent anesthesia. These findings support VenaSeal as a nonthermal endovenous option for the elimination of superficial reflux contributing to venous ulceration as part of comprehensive ulcer care.

## Author Contributions

Conception and design: KG, MG

Analysis and interpretation: KG, MG, RK, NS, DD

Data collection: KG, MG, AK, JS, NN, LO, PG, MK, NK, AD

Writing the article: KG, MG, NS

Critical revision of the article: KG, MG, AK, JS, NN, LO, PG, MK, NK, RK, NS, DD, AD

Final approval of the article: KG, MG, AK, JS, NN, LO, PG, MK, NK, RK, NS, DD, AD

Statistical analysis: NS

Obtained funding: Not applicable

Overall responsibility: KG

KG and MG contributed equally to this article and share co-first authorship

## Data availability

The VenaSeal Spectrum VLU dataset has been publicly available on ClinicalTrials.gov (NCT03820947) since June 2025. The data on which this paper is based are available from the corresponding author (K.G.) upon reasonable request. Trial Registration: VenaSeal Spectrum: Global Post-Market Randomized Controlled Trial, NCT03820947, https://clinicaltrials.gov/study/NCT03820947.

## Funding

The study was sponsored and funded by Medtronic, which contributed to the study design, data collection, and data analysis, and assisted with manuscript writing. The lead authors (K.G. and M.G.) had full access to all study data and had final responsibility for the decision to submit the manuscript for publication. All authors (or their institutions) received research support from Medtronic to conduct this study.

## Disclosures

K.G. serves on the advisory boards of Boston Scientific and Philips and has received research funding from both companies; is a consultant to Gore and has received research funding from it; and is a consultant to Medtronic, has received speaker honoraria and research funding from it. M.G. is a consultant and has received research funding from Medtronic; is a speaker and has received research funding from BD and is an advisory board member and has received research funding from Cook Medical. N.N. has received consulting and speaker fees from Medtronic. L.O. has received research funding from Abbott, Shockwave, and Medtronic and is a consultant for Gore, Huntleigh, and Penumbra. M.K. has received speaker honoraria from Argon Medical, Boston Scientific and Medtronic and is a consultant for Koya Medical. N.K. has received speaker honoraria from Boston Scientific and Medtronic and is a consultant for Boston Scientific. R.K. has received honoraria from Abbott, Auxetics, Koya Medical, Medtronic, Penumbra, Surmodics; serving as a board member of The VIVA Foundation and The Intersocietal Accreditation Council-Vascular Testing; and serving as president of the Syntropic CoreLab. N.S. and D.D. are both employees of Medtronic. A.D. has received grant funding from NIHR, BHF, and Acergy.

## Appendix (online only)

Supplementary Table I

Supplementary Table II

Supplementary Table III

Supplementary Table IV

Supplementary Table V

Supplementary Table VI

Supplementary Table VII
