# Supplementary material for: Twelve-month results of cyanoacrylate closure in patients with venous leg ulceration
Source: J Vasc Surg Venous Lymphat Disord. 2026 Oct 7;15(1):102635. doi: 10.1016/j.jvsv.2026.102635 (PMC13770622; doi:10.1016/j.jvsv.2026.102635)
Supplement: Supplementary file 1 [file mmc1.docx]

**Supplemental Materials**

**Table S1. Spectrum VLU Full Inclusion & Exclusion Enrollment Criteria**

Inclusion Criteria

1. Participant is ≥18 years of age.
2. Participant has venous reflux in superficial truncal vein(s) (e.g., GSV, SSV, ASV, PASV) with CEAP 6 based on the American Venous Forum CEAP classification (2004), appropriate for treatment, as confirmed by DUS.
3. Participants should be eligible for treatment with the VenaSeal™ system.
4. Treatable refluxing segment of target vein(s) 10 cm in length or longer.
5. Participant has a target vein diameter of ≥3 mm throughout the intended treatment segment of the target vein as measured by DUS while participant is standing.
6. Participant is willing and capable of complying with specified follow-up evaluations at the specified times.
7. Participant has the ability to understand the requirements of the study and to provide informed consent.

Exclusion Criteria

1. Participant has a known history of allergic sensitivities (including but not limited to cyanoacrylate adhesives), or any other condition, which in the opinion of the investigator may make the participant more susceptible to cyanoacrylate adhesive hypersensitivity.
2. Participant has known deep vein obstruction in the target limb, as identified by the site’s standard of care.
3. Participant has abnormal pulse exam or ABI <0.8.
4. Participant has acute superficial thrombophlebitis.
5. Participant requires any non-target vein treatments in the contralateral or ipsilateral limb, or any other surgical procedure up to 30 days pre-procedure and through 3 months post-procedure.
6. Participant has any co-morbid conditions, which in the investigator’s opinion may interfere with the participant’s compliance with study visits and procedures, or may confound interpretation of study data (e.g., congestive heart failure Class III and IV, non-ambulatory participants, severe hepatic dysfunction, life expectancy < 1 year).
7. IFU contraindications: Participant has VenaSeal™ system IFU contraindication(s).
8. Participant is non-ambulatory.
9. Participant is a female of childbearing potential who may be pregnant or breastfeeding at the time of the index procedure. *
10. Participant belongs to a vulnerable population per investigator's judgment or participant has any kind of disorder that compromises his/her ability to give written informed consent and/or to comply with study procedures.
11. Participant is currently participating in an investigational drug or device study when the data collected could be conflicting or biased due to participation in another study.
12. Participant has documented COVID-19 infection currently or within the past 3 months. Participant is not completely recovered from past COVID-19 infection, per physician's discretion.
13. Participant has target ulceration identified to be of non-venous etiology, as confirmed by the independent core laboratory.
14. Participant has target circumferential ulceration that cannot be captured in a single photograph (any ulcer curvature around the leg that goes out of sight).

* Pregnancy to be assessed per treating physician routine practice; testing is not required if verbal confirmation is preferred. Breastfeeding participants may be included if mother’s expressed milk is discarded surrounding the index procedure, per the treating physician’s standard instructions. Sites must document routine methods utilized for such participants
