# Supplementary material for: Twelve-month results of cyanoacrylate closure in patients with venous leg ulceration
Source: J Vasc Surg Venous Lymphat Disord. 2026 Oct 7;15(1):102635. doi: 10.1016/j.jvsv.2026.102635 (PMC13770622; doi:10.1016/j.jvsv.2026.102635)
Supplement: Supplementary file 2 [file mmc2.docx]

**Supplementary Materials**

**Table S2. Baseline Participant Demographics and Clinical Characteristics**

| **Characteristic** | **(N=125 Subjects)** |
| --- | --- |
| **Race^a^** |  |
| Not reportable per local laws or regulations | 44.0% (55/125) |
| Subject declines to answer | 0.0% (0/125) |
| unknown | 2.4% (3/125) |
| Black or African American | 2.4% (3/125) |
| Chinese | 0.0% (0/125) |
| Japanese | 0.0% (0/125) |
| Korean | 0.0% (0/125) |
| Native American or Alaska Native | 0.0% (0/125) |
| Native Hawaiian or other Pacific islander | 0.0% (0/125) |
| White | 50.4% (63/125) |
| Other Asian | 0.8% (1/125) |
| Other | 0.0% (0/125) |
| **Lifestyle** |  |
| **Smoking** |  |
| Current | 13.6% (17/125) |
| Former | 34.4% (43/125) |
| Never | 52.0% (65/125) |
| **Baseline physical activity status** |  |
| Does not exercise regularly | 59.2% (74/125) |
| Regular low intensity exercise | 37.6% (47/125) |
| Regular high intensity exercise | 3.2% (4/125) |
| **Subject-reported symptoms** |  |
| Symptoms in the target limb | 92.8% (116/125) |
| Aching | 47.4% (55/116) |
| Burning | 46.6% (54/116) |
| Heaviness | 34.5% (40/116) |
| Itching | 53.4% (62/116) |
| Pain | 65.5% (76/116) |
| Sensitivity | 52.6% (61/116) |
| Swelling | 72.4% (84/116) |
| Throbbing | 28.4% (33/116) |
| Any medications taken^b^ | 72.8% (91/125) |
| **Superficial and Deep Venous Treatment History** |  |
| Deep Venous Stent | 2.4% (3/125) |
| Deep Venous Stent (for pelvic venous disorders) | 0.8% (1/125) |
| Thermal ablation (Laser, RF) | 14.4% (18/125) |
| Non-thermal ablation (MOCA, PEM) | 0.0% (0/125) |
| VenaSeal | 4.0% (5/125) |
| Other cyanoacrylate | 0.0% (0/125) |
| Surgical stripping | 5.6% (7/125) |
| Ultrasound-guided foam sclerotherapy | 4.0% (5/125) |
| **Other Venous History** |  |
| Clotting Disorders | 4.8% (6/125) |
| Deep Vein Thrombosis (DVT) | 17.6% (22/125) |
| Family History of Chronic Venous Disease | 27.2% (34/125) |
| Pelvic Venous Disorder^c^ | 2.1% (1/48) |
| Pulmonary Embolism | 3.2% (4/125) |
| Restless Leg Syndrome | 9.6% (12/125) |
| Venous Claudication | 16.0% (20/125) |
| **Cardiac History** |  |
| Congestive Heart Failure | 11.2% (14/125) |
| Heart Failure with Preserved Ejection Fraction (HFPEF) | 2.4% (3/125) |
| Hyperlipidemia | 36.0% (45/125) |
| Hypertension | 56.0% (70/125) |
| Ischemic Heart Disease | 8.8% (11/125) |
| Previous Myocardial Infarction | 7.2% (9/125) |
| Stroke | 4.0% (5/125) |
| Transient ischemic attack (TIA) | 4.8% (6/125) |
| Other Cardiac | 17.6% (22/125) |
| **Other Disease History** |  |
| Diabetes Mellitus | 18.4% (23/125) |
| History of Obesity | 38.4% (48/125) |
| History of Pregnancy^c^ | 60.4% (29/48) |
| Hypercoagulability Syndrome/Thrombophilia | 3.2% (4/125) |
| Lymphedema | 28.0% (35/125) |
| Peripheral Artery Disease | 11.2% (14/125) |
| Pulmonary Hypertension | 0.8% (1/125) |
| Sleep Apnea | 12.8% (16/125) |
| Vasculitis/Collagen Vascular Disease | 0.0% (0/125) |
|  | |

MOCA: Mechanochemical ablation; PEM: Polidocanol Endovenous Microfoam; RF: Radiofrequency.

^a^Subjects could report one or more races.

^b^Options for reported medication use: Anticoagulants (e.g., Warfarin, Non-vitamin K Antagonist Oral Anticoagulants, Lovenox), antiplatelets / aspirin / P2Y12 Inhibitors, statins, Nonsteroidal anti-inflammatory drugs, immunosuppressants, and other pain medication.

^c^Collected for female subjects only.
