# Supplementary material for: Twelve-month results of cyanoacrylate closure in patients with venous leg ulceration
Source: J Vasc Surg Venous Lymphat Disord. 2026 Oct 7;15(1):102635. doi: 10.1016/j.jvsv.2026.102635 (PMC13770622; doi:10.1016/j.jvsv.2026.102635)
Supplement: Supplementary file 3 [file mmc3.docx]

**Supplementary Materials**

**Table S3. Adjunctive Treatments Through 12 Months**

| **Characteristic** | **N=125 Participants** |
| --- | --- |
| Phlebectomy, rate (N total events) | 0.0 (0) |
| Sclerotherapy, rate (N total events) | 0.12 (19) |

Numbers are rates (count of adjunctive treatments/total vein-years) and total adjunctive treatments. Table demonstrates the total sclerotherapy and phlebectomy used adjunctively on target or perforating veins following the index VenaSeal intervention.
