# Supplementary material for: Twelve-month results of cyanoacrylate closure in patients with venous leg ulceration
Source: J Vasc Surg Venous Lymphat Disord. 2026 Oct 7;15(1):102635. doi: 10.1016/j.jvsv.2026.102635 (PMC13770622; doi:10.1016/j.jvsv.2026.102635)
Supplement: Supplementary file 4 [file mmc4.docx]

**Supplementary Materials**

**Table S4. Proportion of Treated Superficial Reflux in Index Procedures^a^**

| **Index Procedure** | **(N = 157 TVs)** |
| --- | --- |
| Target Veins, n | 153 |
| Mean ± SD, % | 90.8 ± 13.21* |
| Median (Min, Max), % | 98.3 (36.8, 100.0) |

Numbers are presented as a proportion of treated superficial target vein %. Percentages in mean ± standard deviation of (minimum, maximum). TV: Target vein.

*truncal reflux cannot exceed 100

^a^Elimination of clinically relevant superficial truncal disease in each target vein (TV) at the time of index procedure as measured by the percentage of target vein length successfully treated.
