# Supplementary material for: Twelve-month results of cyanoacrylate closure in patients with venous leg ulceration
Source: J Vasc Surg Venous Lymphat Disord. 2026 Oct 7;15(1):102635. doi: 10.1016/j.jvsv.2026.102635 (PMC13770622; doi:10.1016/j.jvsv.2026.102635)
Supplement: Supplementary file 5 [file mmc5.docx]

**Supplementary Materials**

**Table S5: Summary of Return to Work Through 12 Months**

| **Current Working Status at Baseline** | **N=125 Subjects** |
| --- | --- |
| Employed or Independent Worker | 33.6% (42/125) |
| Employed Full Time | 85.7% (36/42) |
| Employed Part Time | 14.3% (6/42) |
| Not Employed | 66.4% (83/125) |
| Seeking Employment | 3.6% (3/83) |
| Not Working by Choice | 80.7% (67/83) |
| Unable to Work Due to Venous Disease | 3.6% (3/83) |
| Unable to Work Due to Other Medical Condition or Disability | 12.0% (10/83) |
| Unknown / Refused to Answer | 0.0% (0/125) |
| **Occupation** |  |
| Sedentary occupation | 12.0% (15/125) |
| Standing occupation | 12.8% (16/125) |
| Walking occupation | 8.8% (11/125) |
| Not applicable | 66.4% (83/125) |
| **Returned** | 95.2% (40/42) |
| At Normal Duties | 75.0% (30/40) |
| At Light Duties | 17.5% (7/40) |
| At Less Than Normal Schedule | 5.0% (2/40) |
| Did Not Respond | 2.5% (1/40) |
| **Not Yet Returned** | 0.0% (0/42) |
| **Exited Before Return to Work Reported** | 4.8% (2/42) |

Summary of activities of VLU participants who underwent VenaSeal intervention through 12 months follow-up. Most patients (95.2%) that were employed returned to normal activities with no limitations (69.0%) following intervention. Numbers are presented as percentage (n/N).
