# Supplementary material for: Twelve-month results of cyanoacrylate closure in patients with venous leg ulceration
Source: J Vasc Surg Venous Lymphat Disord. 2026 Oct 7;15(1):102635. doi: 10.1016/j.jvsv.2026.102635 (PMC13770622; doi:10.1016/j.jvsv.2026.102635)
Supplement: Supplementary file 6 [file mmc6.docx]

**Supplementary Materials**

**Table S6. Venous Dependent Quality-of-Life through 12 Months**

| **Visit** | **(N=125 Subjects)** |
| --- | --- |
| **Baseline** |  |
| n | 125 |
| Quality-of-Life |  |
| Excellent | 6.5% (8/123) |
| Very Good | 17.1% (21/123) |
| Good | 29.3% (36/123) |
| Neither Good Nor Bad | 26.8% (33/123) |
| Bad | 16.3% (20/123) |
| Very Bad | 3.3% (4/123) |
| Extremely Bad | 0.8% (1/123) |
| Predicted QoL without Varicose Veins |  |
| Very Much Better | 42.3% (52/123) |
| Much Better | 38.2% (47/123) |
| A Little Better | 15.4% (19/123) |
| The Same | 4.1% (5/123) |
| Worse | 0.0% (0/123) |
| **Day 30** |  |
| n | 120 |
| Quality-of-Life |  |
| Excellent | 4.2% (5/118) |
| Very Good | 30.5% (36/118) |
| Good | 35.6% (42/118) |
| Neither Good Nor Bad | 20.3% (24/118) |
| Bad | 8.5% (10/118) |
| Very Bad | 0.8% (1/118) |
| Extremely Bad | 0.0% (0/118) |
| Predicted QoL without Varicose Veins |  |
| Very Much Better | 24.6% (29/118) |
| Much Better | 49.2% (58/118) |
| A Little Better | 16.9% (20/118) |
| The Same | 9.3% (11/118) |
| Worse | 0.0% (0/118) |
| **Month 6** |  |
| n | 99 |
| Quality-of-Life |  |
| Excellent | 10.2% (10/98) |
| Very Good | 29.6% (29/98) |
| Good | 35.7% (35/98) |
| Neither Good Nor Bad | 16.3% (16/98) |
| Bad | 6.1% (6/98) |
| Very Bad | 2.0% (2/98) |
| Extremely Bad | 0.0% (0/98) |
| Predicted QoL without Varicose Veins |  |
| Very Much Better | 27.6% (27/98) |
| Much Better | 35.7% (35/98) |
| A Little Better | 12.2% (12/98) |
| The Same | 24.5% (24/98) |
| Worse | 0.0% (0/98) |
| **Month 12** |  |
| n | 87 |
| Quality-of-Life |  |
| Excellent | 9.4% (8/85) |
| Very Good | 29.4% (25/85) |
| Good | 35.3% (30/85) |
| Neither Good Nor Bad | 15.3% (13/85) |
| Bad | 8.2% (7/85) |
| Very Bad | 2.4% (2/85) |
| Extremely Bad | 0.0% (0/85) |
| Predicted QoL without Varicose Veins |  |
| Very Much Better | 22.4% (19/85) |
| Much Better | 25.9% (22/85) |
| A Little Better | 22.4% (19/85) |
| The Same | 28.2% (24/85) |
| Worse | 1.2% (1/85) |

Numbers are presented as percentage (n/N). QoL – quality-of-life.
