# Supplementary material for: Twelve-month results of cyanoacrylate closure in patients with venous leg ulceration
Source: J Vasc Surg Venous Lymphat Disord. 2026 Oct 7;15(1):102635. doi: 10.1016/j.jvsv.2026.102635 (PMC13770622; doi:10.1016/j.jvsv.2026.102635)
Supplement: Supplementary file 7 [file mmc7.docx]

**Supplementary Materials**

**Table S7. Participant or Physician-Reported QoL Improvements through 12 months**

| **Visit** | **Baseline** | **Day 30** | **Month 6** | **Month 12** |
| --- | --- | --- | --- | --- |
| **Instrument, mean (SD) [N]** | | | | |
| EQ-5D Index | 0.57 (0.34) [125] | 0.74 (0.29) [120] | 0.78 (0.27) [98] | 0.78 (0.26) [84] |
| SF-36 physical | 35.54 (8.17) [122] | 37.84 (9.27) [119] | 39.62 (9.76) [97] | 39.88 (9.29) [84] |
| mAVVQ | 33.3 (13.62) [124] | 24.7 (15.46) [115] | 19.9 (14.75) [98] | 18.3 (13.81) [80] |
| rVCSS | 18.5 (4.03) [125] | 12.9 (5.12) [120] | 9.5 (5.50) [99] | 9.2 (5.58) [84] |
| VenousTSQs | NA | 30.9 (4.87) [118] | 32.2 (5.26 [96] | 32.1 (5.32) [84] |

Data is presented as mean (standard deviation) [number of participants]. QoL – quality-of-life, EQ-5D – EuroQoL 5 dimensions standardized quality-of-life survey, SF-36 – short form quality-of-life survey, mAVVQ – modified Aberdeen Varicose Vein Questionnaire, rVCSS – Revised Venous Clinical Severity Score, VenousTSQs – Venous treatment satisfaction questionnaire, NA – not applicable at baseline.
