# Supplementary material for: Twelve-month results of cyanoacrylate closure in patients with venous leg ulceration
Source: J Vasc Surg Venous Lymphat Disord. 2026 Oct 7;15(1):102635. doi: 10.1016/j.jvsv.2026.102635 (PMC13770622; doi:10.1016/j.jvsv.2026.102635)
Supplement: Supplementary file 8 [file mmc8.docx]

**Supplementary Materials**

**Figure S1. Kaplan-Meier Cumulative Incidence of Recurrence Through 12 Months**

Kaplan-Meier curve of freedom from recurrence of ulceration through 12 months in the Spectrum VLU cohort. Ulcer recurrence rate is based on the time between the first ulcer healing and the first ulcer recurrence date. Ulcer healing starts at 0 days.
